# Multiple Regression Analysis of mRNA-miRNA Associations in Colorectal Cancer Pathway

**DOI:** 10.1155/2014/676724

**Published:** 2014-05-07

**Authors:** Fengfeng Wang, S. C. Cesar Wong, Lawrence W. C. Chan, William C. S. Cho, S. P. Yip, Benjamin Y. M. Yung

**Affiliations:** ^1^Department of Health Technology and Informatics, The Hong Kong Polytechnic University, Hong Kong; ^2^Department of Clinical Oncology, Queen Elizabeth Hospital, Hong Kong

## Abstract

*Background*. MicroRNA (miRNA) is a short and endogenous RNA molecule that regulates posttranscriptional gene expression. It is an important factor for tumorigenesis of colorectal cancer (CRC), and a potential biomarker for diagnosis, prognosis, and therapy of CRC. Our objective is to identify the related miRNAs and their associations with genes frequently involved in CRC microsatellite instability (MSI) and chromosomal instability (CIN) signaling pathways. *Results*. A regression model was adopted to identify the significantly associated miRNAs targeting a set of candidate genes frequently involved in colorectal cancer MSI and CIN pathways. Multiple linear regression analysis was used to construct the model and find the significant mRNA-miRNA associations. We identified three significantly associated mRNA-miRNA pairs: BCL2 was positively associated with miR-16 and SMAD4 was positively associated with miR-567 in the CRC tissue, while MSH6 was positively associated with miR-142-5p in the normal tissue. As for the whole model, BCL2 and SMAD4 models were not significant, and MSH6 model was significant. The significant associations were different in the normal and the CRC tissues. *Conclusion*. Our results have laid down a solid foundation in exploration of novel CRC mechanisms, and identification of miRNA roles as oncomirs or tumor suppressor mirs in CRC.

## 1. Introduction

Colorectal cancer (CRC) is a leading cause of cancer-related mortality worldwide with a rapidly increasing incidence in China in the past decade [1]. CRC originates from the accumulation of acquired genetic and epigenetic alterations that lead to the transformation of normal epithelial cells to invasive adenocarcinomas at the cellular level [2, 3]. There are two distinct pathways identified in CRC: (i) microsatellite instability (MSI) pathway with gain or loss of repeat units in a germline microsatellite allele as well as defects in the mismatch repair mechanisms and (ii) chromosomal instability (CIN) pathway with gain or loss of chromosomal regions [4]. Studies of these two pathways have shown that CRC is a genetically heterogeneous disease [4]. It is well known that different patients have different genetics of tumor resulting in heterogeneity with inaccurate prognosis and ineffective treatment when a single biomarker is utilized [5]. As a result, it is desirable to examine the patient's genetic profile so as to more accurately represent the clinical condition of the patient and hence the treatment strategies can be more effective [5].

MicroRNAs (miRNAs) are small single-stranded noncoding RNA molecules about 22 nucleotides long and can posttranscriptionally regulate target gene expression by binding to the 3′ untranslated region of mRNAs [6]. MiRNAs regulate target genes in two ways: repressing the translation of mRNAs to inhibit protein expression or directly degrading mRNAs [7–9]. MiRNA is regarded as a potential biomarker in cancer since it has been found to be chemically stable and also detected in a wide range of clinical samples [10, 11]. Previous studies have proved that some candidate miRNAs can be used as diagnostic, prognostic, or therapeutic biomarkers in CRC. For instance, miR-92a, a potential diagnostic biomarker for early detection of CRC, was reported to be overexpressed in CRC when compared to normal individuals and is known to participate in cell proliferation and apoptosis processes [12]. Overexpression of miR-21 is regarded as a potential predictor of overall survival of CRC patients. MiR-21 probably promotes the invasion and metastasis of tumor by downregulating the expression of Pdcd4, a 64 kDa protein inhibiting tumor progression [12]. MiR-140 was found to be related to multiple drug resistance and was thus a candidate target for CRC therapy to overcome the drug resistance [12].

In this study, we aimed to identify the roles of miRNAs associated with a set of target genes frequently involved in colorectal cancer MSI and CIN signaling pathways. In order to achieve this goal, a regression model was adopted to identify miRNAs that had significant effects on a set of candidate genes. Multiple linear regression analysis of the mRNA-miRNA associations was used to identify the effect of multiple miRNAs on one target mRNA. Target genes are assumed to be negatively associated with the corresponding miRNAs if they are degraded by miRNAs. Positive or negative regression coefficients will demonstrate the promotion or repression effect of an mRNA with the increased miRNA expression, respectively. This bioinformatics study explored the functional role of CRC-pathway-related miRNAs via multiple linear regression analysis in colorectal cancer.

## 2. Methods

### 2.1. Microarray Expression Data

Microarray technology can detect gene expression on a genomic scale. In order to perform the following analysis, we obtained from Gene Expression Omnibus (GEO) repository the processed and normalized microarray expression dataset GSE35982, which analyzed gene expression of 19,135 mRNAs and 851 human miRNAs [13]. The samples were collected from eight CRC tissues and eight matched adjacent normal colorectal mucosa tissues (at least 5 cm from the tumor region). The subjects were treated with surgical excision for CRC at Xinhua Hospital, School of Medicine, Shanghai Jiaotong University [13]. Two inclusion criteria were used to select the subjects: (i) the CRC tissues should have more than 80% tumor cells and (ii) the matched adjacent normal colorectal mucosa tissues should have normal mucosal structure without dysplastic cells [13]. The reason we chose this dataset was that the matched mRNA and miRNA data were collected from the same subjects and were more suitable to explore the difference in the cancer and the normal tissues. In the microarray data, a gene/miRNA may be interrogated by multiple probes. We considered the average expression level of all the probes for the same mRNA/miRNA to handle this situation [14, 15].

### 2.2. Selection of Candidate Genes Frequently Involved in CRC Pathways

MSI and CIN signaling pathways are two major mechanisms of genomic instability in CRC [4]. Important genetic changes have been reported in these two pathways, such as the activation of KRAS and inactivation of p53 in the CIN pathway, as well as the inactivation of the DNA mismatch repair genes MLH1 and MSH2 in the MSI pathway [4]. Moreover, different patients have different genetics of tumor resulting in heterogeneity with inaccurate prognosis and ineffective treatment [5]. Therefore, there is a desperate need to discover more potential targets and it will be useful to examine the dysregulation of genes involved in these two pathways. In this study, we considered genes involved in colorectal cancer MSI and CIN signaling pathways from Kyoto Encyclopedia of Genes and Genomes (KEGG) database (http://www.genome.jp/kegg/) at the first step. And then, we selected candidate genes reported to be frequently involved in CRC from literature among those genes in MSI and CIN signaling pathways. After obtaining the candidate genes, we further extracted the available expression levels of their mRNAs from the microarray dataset GSE35982 to perform the following regression analysis.

### 2.3. Identification of miRNAs Targeting the Candidate Genes

Systematic searches for miRNAs targeting the candidate genes were performed on eight miRNA prediction databases (Table 1). Among these prediction databases, some databases predict the miRNA targets according to some criteria, such as complementarity between miRNA and mRNA, free energy to form the miRNA-mRNA duplex, and conservation among different species as in* TargetScan* [16, 17]. The machine learning approach is also one method to predict the miRNA targets in some databases, such as support vector regression (SVR) in* miRanda-miSVR* database [18]. We obtained the miRNAs targeting each candidate gene from all these eight databases. In order to increase the prediction accuracy, we only selected the miRNAs that were predicted in more than three databases out of all the eight databases. The number of unknown parameters to be estimated in a multiple regression model is the number of selected miRNAs plus one (constant). It is required for each model that the number of independent observations, that is, the samples in a group, is larger than that of unknown parameters so as to maintain the excess of information (degree of freedom). In other words, the number of samples limits the number of miRNAs to be considered in a model. In our study, if the number of selected miRNAs targeting one mRNA plus one was not less than that of samples (eight samples) in the group, we further reduced the selected miRNAs by choosing the ones that were predicted in more than four databases, or even five, six, and so on. After obtaining the list of targeting miRNAs, we extracted the available expression profiles of these miRNAs from the microarray dataset GSE35982.

### 2.4. Multiple Linear Regression Model Analysis

Candidate mRNAs and their targeting miRNAs together with the corresponding expression profiles were considered for further analysis in this part. We investigated the associations of each mRNA transcript with the corresponding miRNAs by means of multiple linear regression analysis based on microarray expression levels (Formula (1)). The 95% confidence interval (CI) was applied to test the significance of each regression coefficient in the regression model. If the 95% CI for a coefficient did not cover zero, there was less than 5% chance (*P* < 0.05) that the coefficient was zero. In other words, this coefficient for a miRNA was significant in the model. IBM SPSS Statistics and MATLAB Statistics Toolbox were used for this analysis. Analysis of variance (ANOVA) of regression was used to demonstrate the significance of the whole model [19]. We applied multiple-hypothesis correction by following Benjamini-Hochberg algorithm to calculate false discovery rates (FDRs) based on *P* values from ANOVA [20]. FDR is used to control the expected proportion of false positives. The FDR value was estimated via the MATLAB function,* mafdr* [21]

(1) $$\begin{array}{c} y=a_{0}+a_{1}x_{1}+a_{2}x_{2}+\cdots +a_{n}x_{n}, \end{array}$$

where *y* represents the expression profile of an mRNA; *x*_1_, *x*_2_,… and *x*_*n*_ represent the expression levels of the corresponding miRNAs targeting the mRNA; *a*_0_ is the regression constant; *a*_1_, *a*_2_,… and *a*_*n*_ are the regression coefficients for each miRNA.

## 3. Results

### 3.1. Identification of Candidate Genes Frequently Involved in CRC Pathways and the Targeting miRNAs

**Figure 1:**
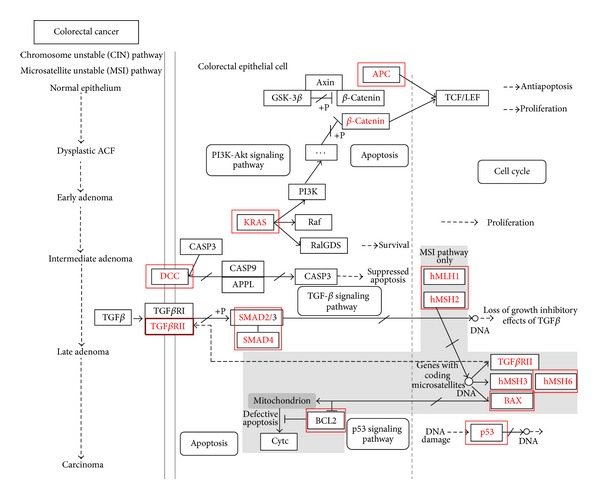
Colorectal cancer MSI and CIN signaling pathways modified from KEGG database. Genes marked with red rectangles are those candidate genes frequently involved in CRC.

In order to discover more potential targets for personalized target therapy, we identified 12 genes that are frequently involved in CRC [4] and defined in KEGG colorectal cancer MSI and CIN signaling pathways for multiple linear regression analysis (Table 2 and Figure 1): Adenomatous polyposis coli (APC); BCL2-associated X protein (BAX); B-cell chronic lymphocytic leukaemia/lymphoma 2 (BCL2); Deleted in colorectal cancer (DCC); Kirsten rat sarcoma viral oncogene homolog (KRAS); MutL homologue 1 (MLH1); MutB homologues 2 and 6 (MSH2 and MSH6); SMAD family members 2 and 4 (SMAD2 and SMAD4); Transforming growth factor, beta receptor II (TGFBR2), and Tumor protein p53 (TP53). For instance, the mutations of APC, KRAS, and TP53 play a vital role in the evolution of colorectal cancer [4]. Besides, all these 12 genes have important molecular functions in the related pathways. Some genes (e.g., MLH1 and MSH2) are involved only in the MSI pathway. Some genes (e.g., APC) function in both MSI and CIN pathways. MiRNAs targeting these candidate genes were searched using the eight miRNA prediction databases. The results are shown in Supplementary Table S1. available online at http://dx.doi.org/10.1155/2014/676724. We further extracted the expression profiles of both mRNAs and the corresponding miRNAs from the microarray dataset GSE35982 for regression analysis.

### 3.2. Multiple Linear Regression Models: Association Analysis between Candidate Genes and the Targeting miRNAs

**Figure 2:**
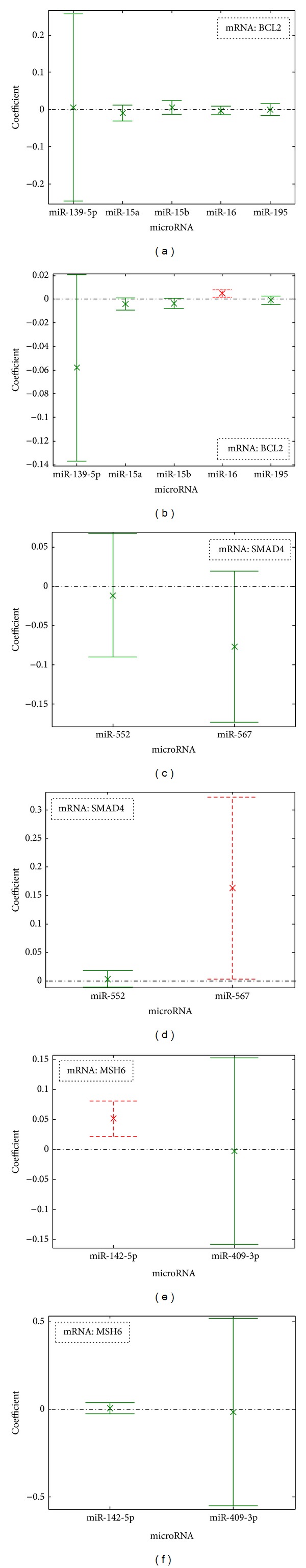
Multiple linear regression analysis: the bar represents the 95% CI for the experimental coefficient estimates. The mark “*x*” is the position for the estimate of each regression coefficient. The significant regression coefficient is marked with dashed red lines and not significant is marked with solid green lines. (a) BCL2 against miRNAs in the normal tissue; (b) BCL2 against miRNAs in the CRC tissue; (c) SMAD4 against miRNAs in the normal tissue; (d) SMAD4 against miRNAs in the CRC tissue; (e) MSH6 against miRNAs in the normal tissue; (f) MSH6 against miRNAs in the CRC tissue.

We investigated the associations of each mRNA transcript of the candidate genes frequently involved in CRC pathways with the corresponding miRNAs via multiple linear regression analysis. Among all these 12 candidate genes, only three genes had significant miRNAs based on 95% CI: BCL2, SMAD4, and MSH6 (Figure 2). The selected miRNAs targeting these three genes are shown in Table 3. The models with ANOVA *P* values less than 0.05 and FDRs less than 0.1 were regarded as significant models. A previous study showed that the FDR values less than 0.3 were selected for their analysis [13]. We used more stringent criteria than their study. In the CRC tissue, two models were found to have significantly associated miRNAs: BCL2 and SMAD4 models. For BCL2 model, the results are shown in Figures 2(a) and 2(b) and Table 4: BCL2 was positively associated with miR-16 (*a* = 0.0047, 95% CI = [0.0015, 0.0079], *P* = 0.0239). For SMAD4 model, the results are shown in Figures 2(c) and 2(d) and Table 4: SMAD4 was positively associated with miR-567 (*a* = 0.1624, 95% CI = [0.0031, 0.3218], *P* = 0.0471). However, these two models were not significant as a whole: ANOVA *P* values = 0.083 and 0.103, FDRs = 0.506 and 0.314. In the normal tissue, the MSH6 model was identified (Figures 2(e) and 2(f) and Table 5): MSH6 was positively associated with miR-142-5p (*a* = 0.0514, 95% CI = [0.0217, 0.0811], *P* = 0.0067). Most importantly, the whole MSH6 model was significant: ANOVA *P* value = 0.018, FDR = 0.080.

## 4. Discussion and Conclusion

In this study we explored the mRNA-miRNA associations in colorectal cancer MSI and CIN signaling pathways by means of multiple linear regression analysis. Three significantly associated mRNA-miRNA pairs were found: BCL2 with miR-16 and SMAD4 with miR-567 in the CRC tissue, and MSH6 with miR-142-5p in the normal tissue. As for the whole model, BCL2 and SMAD4 models were not significant, and MSH6 model was significant. Combining miRNA targets from prediction databases with regression analysis, we can further eliminate the false positive for the identification of miRNAs. More important is that we can predict how many miRNAs work together on the same mRNA and what the effect direction of each miRNA on the mRNA is. In this study, only one significant miRNA was identified in each model. Sometimes, more than one miRNA would be found. Ultimately we can identify the normal tissue specific or CRC tissue specific miRNAs, which will be helpful for the diagnosis and treatment of CRC.

The results showed that BCL2 was positively associated with miR-16, and SMAD4 was positively associated with miR-567 in the CRC tissue (Figure 2 and Table 4). In the normal tissue, we found that MSH6 was positively associated with miR-142-5p (Figure 2 and Table 5). In this study, only positive associations were found between miRNAs and mRNAs. However, sometimes negative associations can also be found. MiRNAs can affect the expression of mRNAs in two directions: positive and negative. From the literature, we know that miRNAs have two important functions: repressing the translation of mRNAs to inhibit protein expression, and directly degrading mRNAs [7–9]. The target genes are assumed to be negatively correlated with the corresponding miRNAs if the target genes are degraded by miRNAs [22, 23]. It has been found that the binding of miRNAs to its target mRNAs can lead to the cellular accumulation of the inhibited mRNAs in plant [23, 24], which may further lead to the positive association between mRNA and the corresponding miRNA as shown in our study.

One miRNA (miR-16) was found to be significantly associated with its target gene (BCL2) in the CRC tissue in our study. BCL2 is a protooncogene, coding a 26 kd protein, involved in the regulation of cell death by inhibiting apoptosis, which is often overexpressed in CRC [25]. However, BCL2 functions only in the situation where apoptosis is needed for development or cell renovation in the normal tissues [26]. As a result, the phenotypic expression of BCL2 is a useful prognostic molecular marker in colorectal adenocarcinoma [27]. It has been reported that BCL2 can be regulated by miR-16 in a leukemic cell line model and breast cancer cells [28, 29]. In the CRC tissue, we also found that SMAD4 was positively associated with miR-567. SMAD4 is a major component of the transforming growth factor *β* signaling pathway, which is mutated in approximately 15% of colorectal cancers [30]. Moreover, the allelic loss containing chromosome 18q, the loci of SMAD2 and SMAD4, was found in most colorectal cancers [31]. These two significant mRNA-miRNA associations can be found only in the CRC tissue. MiR-16 and miR-567 can be regarded as oncomirs that tightly regulate BCL2 and SMAD4 expression in CRC, which will be helpful for the diagnosis and treatment of CRC.

Moreover, miR-142-5p was found to have significant association with MSH6 from the regression model in the normal tissue. MSH6 proteins function in the MSI pathway, resulting from the germ-line mutation in the mismatch repair mechanisms [4]. The mutations found in MLH1, MSH2, and MSH6 genes account for the majority of DNA mismatch repair defects [32]. These mRNA-miRNA associations can be found only in the normal tissue but not in the cancer tissue. Hence, lack of this association is a possible reason for the dysregulation of proliferation and apoptosis, as well as the defects in DNA mismatch repair in CRC. MiR-142-5p can be regarded as a tumor suppressor mir in the normal tissue. Since the whole MSH6 model was significant, it can be regarded as a signature for the normal tissue.

Compared to Pearson correlation analysis to indicate the relationship between miRNAs and mRNAs, our study introduced multiple linear regression analysis, which is more suitable for studying their relationship and mimicking the real situation* in vivo*. Multiple miRNAs targeting the same mRNA may function together during gene expression process. Moreover, we selected the targeting miRNAs through more than one miRNA target prediction database, which makes the prediction accuracy more reliable. A previous study of Li et al. showed that miRNAs included in at least three of four prediction databases were chosen [33]. In our study, we applied more miRNA prediction databases (eight databases) and the selected miRNAs were predicted in at least four databases when comparing with their study.

Several similar algorithms have been proposed to study the mRNA-miRNA associations. In Lu et al.'s study, a lasso regression model was proposed by combining miRNA target prediction information, miRNA coregulation, and RNA-induced silencing complexes (RISC) availability [34]. In that model they thought that argonaute protein involved in RNA-induced silencing complex may affect the expression of mRNAs, which was also included in the mRNA-miRNA association model [34]. In Beck et al.'s study, they constructed the regression model including both miRNAs and transcription factors to predict mRNA expression level [35]. In our study, we were only interested to explore what the effect of multiple miRNAs on the expression levels of mRNAs is. Compared to these studies, our study has the following features: (i) we focused on a particular disease and extracted some important genes on a particular pathway to explore the mechanisms of the disease and (ii) we selected the targeting miRNAs through eight target prediction databases, which makes the prediction accuracy more reliable. While in their studies only two databases were chosen. The number of samples limits the number of miRNAs to be considered in a multiple regression model. It is possible to select more miRNAs for the model if microarray data of more samples are used.

In summary, we have presented a detailed regression model to explore the mRNA-miRNA associations in the normal and the CRC tissues. This bioinformatics study is capable of exploring the functional role of CRC-pathway-related miRNAs via multiple linear regression analysis in cancer research, which can also identify specific miRNAs function as oncomirs or tumor suppressor mirs. Finally, the results generated from this study will be helpful in the diagnosis and treatment of CRC.

## Supplementary Material

Supplementary Table S1: presents a list of candidate genes frequently involved in CRC pathways and their potential targeting microRNAs. The targeting microRNAs were selected as the independent variables of the regression models based on the criterion that they are predicted by more than 3 out of the eight prediction databases.

## Figures and Tables

**Table 1:** Prediction databases for identifying targeting miRNAs.

Database name	Species	Website
miRDB	Human, mouse, rat, dog, chicken	http://mirdb.org/miRDB/
miRWalk	Human, mouse, rat	http://www.umm.uni-heidelberg.de/apps/zmf/mirwalk/predictedmirnagene.html
PicTar	Vertebrates, flies	http://pictar.mdc-berlin.de/
TargetScan	Any	http://www.targetscan.org/
DIANA-microT	Any	http://diana.cslab.ece.ntua.gr/microT/
MicroCosm-Targets	Any	http://www.ebi.ac.uk/enright-srv/microcosm/htdocs/targets/v5/
miRanda-mirSVR	Any	http://www.microrna.org/microrna/getGeneForm.do
miRecords	Any	http://mirecords.biolead.org/prediction_query.php

**Table 2:** Candidate genes frequently involved in CRC pathways (modified from [4]).

Gene name	Molecular function	CRC pathway
APC	Tumor suppressor gene	MSI and CIN
BAX	Apoptosis	MSI
BCL2	Antiapoptosis	MSI
DCC	Tumor suppressor gene	MSI and CIN
KRAS	Oncogene	MSI and CIN
MLH1	DNA mismatch repair	MSI
MSH2	DNA mismatch repair	MSI
MSH6	DNA mismatch repair	MSI
SMAD2	Tumor suppressor	MSI and CIN
SMAD4	Tumor suppressor	MSI and CIN
TGFBR2	Cell signaling	MSI and CIN
TP53	Tumor suppressor	MSI and CIN

**Table 3:** The selected miRNAs targeting BCL2, SMAD4, and MSH6.

mRNA	miRNAs	DIANA-microT	MicroCosm-Targets	miRWalk	TargetScan	miRanda-mirSVR	miRDB	PicTar	miRecords
BCL2	hsa-miR-139-5p	*✓*	*✓*	*✓*			*✓*		*✓*
	hsa-miR-15a	*✓*		*✓*		*✓*		*✓*	*✓*
	hsa-miR-15b	*✓*		*✓*		*✓*		*✓*	*✓*
	hsa-miR-16	*✓*		*✓*		*✓*		*✓*	*✓*
	hsa-miR-195	*✓*		*✓*		*✓*		*✓*	*✓*

SMAD4	hsa-miR-552	*✓*	*✓*	*✓*			*✓*		*✓*
	hsa-miR-567	*✓*	*✓*	*✓*			*✓*		*✓*

MSH6	hsa-miR-142-5p		*✓*		*✓*		*✓*		*✓*
	hsa-miR-409-3p		*✓*		*✓*		*✓*		*✓*

**Table 4:** Multiple linear regression analysis for BCL2 and SMAD4 in the CRC tissue.

mRNA	FDR	miRNAs	Coefficient (*a*)	95% CI	*P *
BCL2	0.506	hsa-miR-139-5p	−0.0580	[−0.1368, 0.0208]	0.0870
		hsa-miR-15a	−0.0042	[−0.0095, 0.0012]	0.0779
		hsa-miR-15b	−0.0036	[−0.0081, 0.0008]	0.0719
		**hsa-miR-16**	0.0047	[0.0015, 0.0079]	***0.0239***
		hsa-miR-195	−0.0009	[−0.0046, 0.0027]	0.3837

SMAD4	0.314	hsa-miR-552	0.0034	[−0.0113, 0.0180]	0.5822
		**hsa-miR-567**	0.1624	[0.0031, 0.3218]	***0.0471***

**Table 5:** Multiple linear regression analysis for MSH6 in the normal tissue.

mRNA	FDR	miRNAs	Coefficient (*a*)	95% CI	*P *
MSH6	***0.080***	**hsa-miR-142-5p**	0.0514	[0.0217, 0.0811]	***0.0067***
		hsa-miR-409-3p	−0.0033	[−0.1587, 0.1521]	0.9585
